# New strategies for energy supply of cardiac implantable devices

**DOI:** 10.1007/s00399-022-00852-0

**Published:** 2022-04-04

**Authors:** Caroline Moerke, Anne Wolff, Hüseyin Ince, Jasmin Ortak, Alper Öner

**Affiliations:** Department of Cardiology, Rostock University Medical Center, Rostock, Germany

**Keywords:** Cardiovascular implantable electronic device, Battery, Self-powered devices, Energy harvesting, Power supply, Kardiovaskuläres implantierbares elektrisches Gerät, Batterie, Autonome Geräte, Energiegewinnung, Stromversorgung

## Abstract

**Background:**

Heart disease and atrial fibrillation are the leading causes of death worldwide. Patient morbidity and mortality associated with cardiovascular disease can be reduced by more accurate and continuous diagnostic and therapeutic tools provided by cardiovascular implantable electronic devices (CIEDs).

**Objectives:**

Long-term operation of CIEDs continues to be a challenge due to limited battery life and the associated risk of device failure. To overcome this issue, new approaches for autonomous battery supply are being investigated.

**Results:**

Here, the state of the art in CIED power supply is presented and an overview of current strategies for autonomous power supply in the cardiovascular field is given, using the body as a sustainable energy source. Finally, future challenges and potentials as well as advanced features for CIEDs are discussed.

**Conclusion:**

CIEDs need to fulfil more requirements for diagnostic and telemetric functions, which leads to higher energy requirements. Ongoing miniaturization and improved sensor technologies will help in the development of new devices.

## Abstract (de)

**Hintergrund:**

Herzerkrankungen und Vorhofflimmern sind weltweit die häufigsten Todesursachen. Die mit Herz-Kreislauf-Erkrankungen verbundene Morbidität und Mortalität der Patienten lässt sich durch genauere und kontinuierliche Diagnose- und Therapiemöglichkeiten, die durch kardiovaskuläre implantierbare elektronische Geräte („cardiovascular implantable electronic devices“ [CIED]) ermöglicht werden, verringern.

**Fragestellung:**

Der Langzeitbetrieb von CIED ist aufgrund der begrenzten Batterielebensdauer und der damit verbundenen Gefahr eines Geräteausfalls nach wie vor problematisch. Um diese Problematik zu lösen, werden neue Ansätze für eine autonome Stromversorgung untersucht.

**Ergebnisse:**

In dieser Übersicht wird der Stand der Technik der CIED-Stromversorgung dargestellt und ein Überblick über aktuelle Strategien zur autonomen Stromversorgung im kardiovaskulären Bereich gegeben, bei denen der Körper als nachhaltige Energiequelle genutzt wird. Abschließend werden zukünftige Herausforderungen und Potenziale sowie fortschrittliche Funktionen für CIED diskutiert.

**Schlussfolgerung:**

CIED müssen hohe Anforderungen hinsichtlich der Diagnose- und Telemetriefunktionen erfüllen. Dies führt zu einem hohen Energiebedarf. Miniaturisierung und verbesserte Sensortechniken können zur Entwicklung neuer Geräte beitragen.

## Introduction

In Germany, cardiovascular diseases represented 55.9% of all deaths in 2018 and were responsible for more than half of the top 10 causes of death [1]. Therefore, early diagnosis and treatment are crucial for patient survival.

**Fig. 1:**
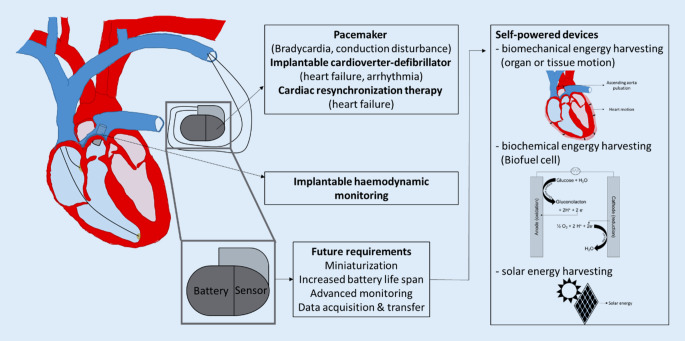
Implementation path of implantable cardiovascular electronic devices with their functions and future requirements as well as an overview of the strategies for self-powered devices

In recent decades, cardiovascular implantable electronic devices (CIEDs) such as pacemakers, implanted cardioverter defibrillators (ICDs), or cardiac monitoring devices have reduced the morbidity and mortality associated with cardiovascular disease by providing more accurate and continuous diagnostic and therapeutic options [44]. However, in the field of CIEDs, new strategies to reduce device size as well as extend battery life and durability are still required (Fig. 1).

## Standard batteries

The first implanted pacemaker by Ake Senning in 1958 was equipped with a rechargeable nickel-cadmium battery bearing a cell voltage of 1.25 V and a capacity of 190 mAh. However, secondary batteries (rechargeable batteries) have a lower capacity compared to primary batteries (non-rechargeable batteries) and consequently have a short life span. Early pulse generators were powered by series-wired mercury-zinc batteries with between three and six cells in series, providing an output voltage of 4–8 V. However, mercury-zinc batteries released hydrogen as a discharge and therefore required venting, which the hermetically sealed devices could not provide to prevent fluid leakage. Mercury-zinc batteries are no longer in use today. For a time, nuclear batteries were also tested. Despite their high toxicity, nuclear batteries had an extended operating life of over 30 years, but also a large volume. With the introduction of the lithium-iodine battery in 1975, the lifespan of pacemaker batteries could be extended considerably, as lithium has a higher energy density and the shelf-life corresponds to a capacity loss of 10% over 5 years [29, 36].

The essential factors for CIEDs are voltage (minimum, maximum), discharge current (initial, average, maximum), size and duration of the current pulses (continuous or intermittent operation), high specific energy and power, long shelf-life and the ability to perform well under varying environmental conditions (temperature, pressure). Finally, the battery of a CIED must accomplish many requirements, e.g. be biocompatible, corrosion resistant, hermetically sealed, lightweight, flat and small and of course reliable. In general, lithium solid cathode primary batteries are used to power advanced implantable medical devices as well as CIEDs since they meet the requirements for voltage characteristics, good longevity, low drain current or self-discharge, high energy density and small size. Lithium liquid cathode systems can provide higher discharge rates but are not suitable for implanted medical devices due to their rapid discharge. Discharge at a solid cathode implies diffusion of lithium ions into the bulk of the cathode, which is a slower process than deposition of discharge products at the liquid cathode. The typical solid cathode materials are manganese dioxide (MnO_2_), copper (II) oxide (CuO), vanadium oxide (V_2_O_5_) and carbon monofluoride (CF)_n_. During the life of a battery, the impedance changes from 50–100 Ω to 20,000–30,000 Ω and the current cannot flow easily through the cell. When current flows from the negative lithium anode to the positive cathode, the lithium reacts with the iodine to form lithium iodine, which expands its volume and enhances resistance. This reaction can be reversed by using more concentrated active materials and increasing the surface area of the anode [29]. CIEDs have a peak power demand of 100–200 µW, which can be maintained by lithium-iodine batteries even with an internal resistance of several thousand ohms. The construction of a lithium-iodine battery includes a single, central lithium anode surrounded by a cathode material that is 96% iodine and thermally fixed with a polymer material to create a conductive mixture. Most of the battery volume is occupied by the central anode with the embedded current collector wire and the iodine cathode. An electrical feedthrough connects the anode to the outside of the cell and the body serves as an electrical connection to the cathode [36].

**Fig. 2:**
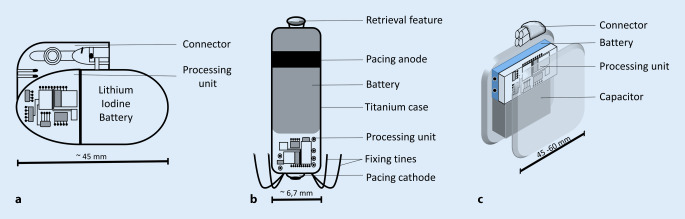
Schematic illustrations of **a** pacemaker device, **b** leadless pacemaker and **c** defibrillator

The power requirements of CIEDs vary widely (Table 1). Cardiac pacemakers require only small amounts of energy, of the order of 15 µJ, resulting in an annual power consumption of 10–100 µW (0.5–2 Ah over 5–10 years), which implies a lifetime of a battery with 1 Ah capacity of 10 years. However, pacemaker battery life depends on both frequency of use and functions, e.g. biventricular pacing with three electrodes consumes more energy than pacing with only one electrode. On the other hand, defibrillators need to generate up to 40 J when providing a defibrillation shock, which leads to a much higher peak energy demand with a shorter battery life (only 4–6 years) compared to the pacemaker [17, 22, 37, 40]. The high-current pacing pulse delivered by the ICD is from a capacitor that is recharged between pacing pulses by converting the low-voltage energy delivered by the battery into high-voltage energy [36]. Therefore, the batteries must be able to rapidly charge the capacitors for the purpose of delivering high-current pulses in the 2–3 A range. Analogous to the pacemaker, the dual chamber ICD requires more energy, resulting in a shorter lifetime compared to single-chamber ICDs. Finally, the most energy-intensive CIEDs are cardiac resynchronization therapy (CRT-D) defibrillators, as they continuously stimulate biventricularly and the stimulation thresholds in the left ventricle are higher [2]. The battery approximately occupies 60%–75% of the volume of a CIED (for schematic illustration see Fig. 2a–c) and the trend should be towards smaller devices that offer greater comfort for patients. New approaches with an energy harvester should also not exceed the current volume [40].

**Table 1:** Summary of the essential factors, requirements and energy demands for CIEDs

Essential factors for a CIED	Requirements for CIED batteries	CIED energy demands
Voltage (minimum, maximum)	Biocompatible	Pacemaker: 15 μl per stimuli battery life span: 10–12 years
Discharge current (initial, average, maximum)	Hermetically sealed	ICD: 40 J per defibrillation battery life span: 4–6 years
Size and duration of the current pulses (continuous or intermittent operation)	Corrosion resistant	CRT-D: defibrillation + pacing battery life span: 4–6 years
High specific energy, long shelf-life	Light-weighted, flat and small	
Reliable performance under varying conditions	Low drain current or self-discharge	
	High energy densities	

*CRT‑D* Cardiac resynchronisation therapy defibrillators, *CIED* cardiovascular implantable electronic device, *ICD* implanted cardioverter defibrillators

## Biomechanical energy harvesting

A sustainable source of power is the conversion of energy from the human body into electricity. Two approaches have been reported in the literature that performed well in vivo: triboelectric nanogenerators (TENGs) and piezoelectric nanogenerators (PENGs).

**Fig. 3:**
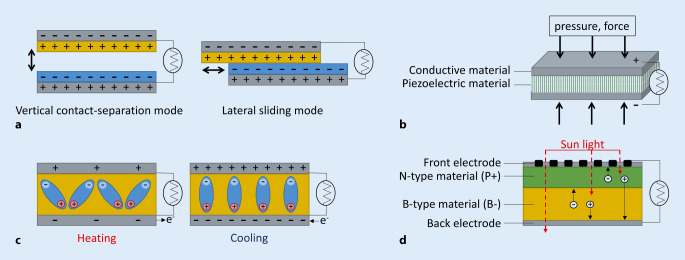
Schematic illustration of the principle of **a** triboelectric nanogenerators, **b** piezoelectric nanogenerators, **c** pyroelectric nanogenerators and **d** solar cells

TENGs are based on the method of mechanical-electrical energy conversion. The nanogenerator consists of two materials with different electron capture properties that, after contact and separation, can carry different charges and eventually generate an electric potential. Under mechanical stress, a constantly changing current output is generated, and this triboelectric potential can drive free electrons into an external circuit to power electronic devices. Traditional TENGs are based on the vertical contact-separation method, where two friction layers are brought into contact vertically and separated again. The electrodes are on the back of the friction layers and connected to an external circuit. In addition to the TENG with vertical contact separation, there is also a TENG with lateral sliding, in which two friction layers both align and slide parallel until the two ends no longer overlap and are separated. An electric field is generated by the spatial distribution of the triboelectricity charges (Fig. 3a). The electrodes of the friction layers are also connected to an external circuit [10].

The first pacemaker TENGs were based on energy harvesting from respiratory movements and were tested on rats by implantation under the left thoracic skin. After implantation, a working surface of 0.8 × 0.8 cm produced an open circuit voltage (V_oc_) of 3.75 V and a short circuit current (I_sc_) of 0.14 µA [43]. A 30 × 20 × 1 mm TENG implanted in the space between the pericardium and epicardium of a pig gave a V_oc_ of 10 V and an I_sc_ of 4 µA. The device also provided monitoring of ventricular premature contractions and atrial fibrillation. The pressure and velocity of blood flow can be estimated from the output voltage [28]. Ouyang et al. demonstrated a fully implanted symbiotic pacemaker based on a TENG that both harvests and stores energy and is a pacemaker for correcting sinus arrhythmias in a large animal model [30]. The TENG generated a maximum V_oc_ of 65.2 V and energy of 0.495 µJ during each cardiac motion cycle, which is above the endocardial pacing threshold energy of 0.377 µJ. In a mongrel in vivo model, a five-staged TENG was shown to charge a lithium-ion battery by harvesting biomechanical body and gravity motion of a subcutaneous implantation site and enable ventricle pacing and sensing operation mode of the self-rechargeable cardiac pacemaker system [31]. TENGs can produce high voltage outputs but the gap between the friction layers needs to be protected from body fluids, otherwise it will significantly affect the output performance. Thus, the TENG must have a biocompatible and flexible design but the size and thickness must be adapted to the available space inside. Finally, the TENG must be sensitive enough to detect even small movements such as breathing or heart contraction [35].

Energy harvesting with PENGs is based on the piezoelectric effect, in which an internal electrical charge is generated by applying mechanical force to a piezoelectric material. Piezoelectric materials have a crystalline structure without inversion symmetry, which enables a linear electromechanical interaction between the mechanical and electrical states. The application of mechanical force results in displacement of anions and cations, creating an electrical dipole moment and potential distribution that triggers a flow of electrons in an external circuit that can be used to power an electronic device (Fig. 3b; [12]). Materials with piezoelectric properties are inorganic materials such as zinc oxide, zirconate titanate (PZT), barium titanate, barium niobate and metaniobate, but some organic materials such as polyvinyl chloride and polyvinylidene fluoride also have piezoelectric properties. Zinc oxide and zirconate titanate are mostly used for the construction of PENGs [44]. In 2010, the first PENG was shown to generate energy from breathing and heart motions in rats [24]. In 2010, the first PENG in rats was shown to generate energy from respiratory and cardiac movements [24]. In the first large animal study in 2014, a PENG implanted in a pig heart, fixed from the left ventricular apex to the right ventricle, was able to achieve a peak-to-peak voltage of 3 V [27], which was increased to 17.8 V in 2017 [19]. When comparing TENGs and PENGs, PENGs are more robust and resistant to long-term repetition of mechanical deformation, but the power output is higher for TENGs. However, TENGs need to be protected from liquid, otherwise the power output will drop significantly [23]. The electrical performance of the nanogenerators has increased over time and Li et al. [22] were able to develop a high-performance PENG with an elastic skeleton and two piezoelectric composites, also outperforming the TENGs. See Table 2 for a comparison of TENG and PENG tested in vivo.

**Table 2:** List of triboelectric nanogenerators (TENGs) and piezoelectric nanogenerators (PENGs) tested in vivo and reported in the literature

Type	Material	Power	Harvesting source	Reference
TENG	PDMS Kapton Au-layer	V_oc_ 3.75 V I_sc_ 0.14 µA → 0.525 µJ	Breathing motion in rat	[43]
TENG	PTFE Kapton Au-layer	V_oc_ 10 V I_sc_ 4 µA → 40 µJ	Heart motion in pig	[28]
TENG	PTFE Kapton Au-layer Al-layer	V_oc_14 V I_sc_ 5 µA → 70 µJ	Heart motion in pig	[45]
TENG	PDMS Ti Kapton Au PTFE Al	V_oc_ 65.2 V I_sc_ 0.5 µA → 32.6 µJ	Heart motion in pig	[30]
TENG	PDMS PTFE Kapton Au-layer Al-layer	V_oc_ 0.008 V	Heart motion in pig	[26]
TENG	Amine-functionalized poly(vinyl alcohol) (PVA-NH2) perfluoroalkoxy (PFA)	V_oc_ 3.75 V → 4.9 µW/cm^3^	Body motion and gravity in mongrel	[31]
PENG	ZnO nanowire on polyimide substrate	V_oc_ 3 mV I_sc_ 30 pA → 0.06 pJ	Breathing and heart motion in rat	[24]
PENG	PZT: Pb(Zr_0.52_Ti_0.48_)O_3_/Pt/Ti/SiO2 Ti/Pt, Cr/Au electrodes	V_oc_ 1 mV I_sc_ 1 pA → 0.001 pJ	Heart motion in rabbit	[4]
PENG	PZT: (Pb(Zr_0.52_Ti_0.48_)O_3_) Ti/Pt electrodes, SiO_2_, Si on polyimide substrate	V_oc_ 3 V	Heart motion in pig	[27]
PENG	PZT: Pb(Mg_1/3_Nb_2/3_)O_3_− xPbTiO_3_ Au electrodes On PET substrate	V_oc_ 8.2 mV I_sc_ 0.223 mA → 1.8286 µJ	Heart motion in rat	[17]
PENG	PVDF Al electrodes	V_oc_ 1.5 mV I_sc_ 0.3 mA at 160/105 mm Hg → 0.45 µJ	Blood pressure ascending aorta in pig	[42]
PENG	PZT: Pb(Mg_1/3_Nb_2/3_)O_3_− (x)Pb(Zr,Ti)O_3_ on PU and PET substrate	V_oc_ 17.8 V I_sc_ 1.74 µA → 30.972 µJ	Heart motion in pig	[19]
PENG	PZT: Pb(Mg_1/3_Nb_2/3_)O_3_-(28%)-PbTiO_3_ Cr/Au, Be electrodes on PDMS substrate	V_oc_ 20 V I_sc_ 15 μA → 300 µJ	Heart motion in pig	[22]
PENG	PMN-Pt ((72%) Pb (MGI/3NB2/3)O3− (28%) PbTiO3, 300 μm, TrSx2A, TrS Ceramics)	V_oc_ 3.2 V I_sc_ 54 nA	Heart motion in rat	[38]

*Al* Aluminium, *Be* Beryllium, *Cr* Chromium, *Au* Gold, *Pb* Lead, *PZT* Lead zirconate titanate, *PENG* Piezoelectric nanogenerator, *PMN-Pt* Polycrystalline lead magnesium niobate–lead titanate, *PDMS* Polydimethylsiloxane, *PET* Polyethylene terephthalate, *PTFE* Polytetrafluoroethylene, *PU* Polyurethane, *PVDF* Polyvinylidene fluoride, *Si* Silicon, *SiO*_*2*_ Silicon dioxide, *xPbTiO*_*3*_ Ternary perovskite, *Ti* Titanium, *TENG* Triboelectric nanogenerator, *ZnO* Zinc oxide, *Zr* Zirconium

For biomechanical energy harvesting, mainly heart beats, blood pressure gradients and arterial wall deformation have been investigated as sources. The human heart beat causes deformation of the myocardium with a frequency of 1–3 Hz, depending on the personʼs activity. The induced strain on the myocardium is 15–23% in the radial and 9–12% in the circumferential direction [8]. The blood pressure gradient varies between 20–100 mm Hg (2.7–13.3 kPa) in the right and left ventricles. The arterial system changes ~ 40 mm Hg (5.3 kPa) and the arterial wall deformation between 15.8 mm (at 118 mm Hg) and 17.3 mm (at 63 mm Hg) at a heart rate of 66 bpm [21, 40]. The diameter distension of the carotid artery is 10% and for the brachial artery 3.7% between diastolic and systolic period [5]. These are sufficient biomechanical actions of the biological system to provide energy. Blood flow is also being investigated as a possible source for energy harvesting, but the risk of blood cell damage and thrombus formation is too high [40].

Another approach to harvesting kinetic energy from the heart is oscillation generators. The first device based on a quartz clock was implanted on the right ventricular wall of a dog and was able to store 80 mJ over 30 min, equivalent to 13 pJ per heartbeat [13]. Others tested a mass imbalance oscillation attached to a sheep heart and achieved an output power of 16.7 µW and 11 µJ per heartbeat, which is sufficient to power a pacemaker [47]. However, these devices had the disadvantage of heavy mass (16.7 g) and size. In 2017, the oscillation generator was improved in design with a smaller mass (7.7 g) and size (radius 3.8 mm). After implantation at an epicardial site in a pig, it was able to generate over 6 µW from the low frequency vibrations [46]. In 2020, the same group published a mass imbalance electromagnetic oscillation generator for the powering of a leadless pacemaker. The device was miniaturized to a volume of 1.2 cm^3^ to provide a catheter-based implantation into the apicoseptal position in the right ventricle of a pig. The average output power during the in vivo tests was 2.6 µW, enough energy to power a leadless pacemaker. But the long term effects on the harvester performance due to encapsulation and mechanical degradation, as well as the impact of the device on the heart muscle, are not yet investigated [11].

## Thermic and biochemical energy harvesters

Biomechanical energy harvesting attracted a lot of attention, but there are other approaches to energy harvesting.

Pyroelectric nanogenerators (PyENGs) can collect energy by converting thermal energy into electric energy via nanomaterials with pyroelectric effects. Pyroelectricity is defined as the temperature-dependent spontaneous polarization in certain anisotropic crystals. Pyroelectric materials have a unique polar axis along with spontaneous polarization exits that create a dipole moment and electrical current/potential during temporal temperature changes (Fig. 3c; [25]). At constant temperatures, no pyroelectric current is generated as the polarization intensity of the materials crystal structure remains unaffected. Connecting the pyroelectric material to an external circuit, cycles of heating and cooling can generate pyroelectric energy powering the circuit [44]. Materials with pyroelectric properties include triglycine sulfate, polyvinylidene fluoride (PVDF), gallium nitride (GaN) as well as zinc oxide (ZnO) and lithium tantalite (LiTaO_3_). The latter two also exhibit piezoelectric properties [25]. Pyroelectric materials such as PVDF are already used in CIED leads to protect them from localized heating, e.g. from radio frequency energy by adsorbing the heat and converting it into electrical energy [18]. Sultana et al. [34] developed a piezoelectric-pyroelectric nanogenerator based on methylammonium lead iodide (CH_3_NH_3_PbI_3_) incorporated in electrospun PVDF nanofibers that can harvest mechanical and thermal energy. To generate a V_oc_ of 42 V and an I_sc_ of 2.5 µA with a PyENG, temperature fluctuations of 5 °C in short time periods are necessary [39]. These temperature–time gradients are not present in the human body, so PyENGs are probably not practical as energy generators for CIEDs.

Biochemical harvesters, such as biofuel cells, are another strategy for generating energy from the body. Biofuel cells convert chemical energy from molecules present in the biofliuds into electrical energy using enzymes as catalysts. In contrast to classical batteries, the reactants for the biofuel cells are constantly provided by the biosystem. Theoretically, biofuel cells are suitable for unlimited operation as long as the reactant or biofuel are supplied. Glucose is the reactant most often used for biofuel cells as it is present in most biofluids [44]. These glucose-powered biofuel cells are based on enzyme electrodes that utilize glucose oxidase for glucose oxidation and laccase for dioxygen reduction (Fig. 1).

Implanted in the abdominal cavity of a rat, these biofuel cells produce a V_oc_ of 0.57 V and a power output of 38.7 mW [41]. A glucose-powered biofuel cell located in the abdominal cavity of a rabbit was shown to function for 2 months. During this time, it wirelessly charged through 100 kΩ load and delivered 16 mW/ml during the 30-min charging time. However, the power output decreased over time due to inflammatory processes [9]. Biofuel cells have a lower power output compared to other energy sources owing to slow electron transfer. However, a glucose oxidase anode assembled layer-by-layer in a biofuel cell exhibited a high power density of 3.7 mW/cm^2^ compared to the usual range of ~ 10 µW/cm^2^ [20]. Another biofuel cell was shown to power a pacemaker ex vivo using a biofuel solution mimicking the human blood circulatory system. These cells produced a V_oc_ of 350 and 470 mV and an I_sc_ of 2.6 as well as 5 mA [16, 33]. The major challenges of biofuel cells are their long-term operational stability, which depends on the lifetime of the enzyme and cofactors, as well as the deterioration and biofouling of the electrodes. The catalytic activity and stability of the enzymes strongly depend on the physiological conditions (pH, temperature, chlorine concentration) of the biofluid [20].

## Solar energy harvesters

Sunlight is the most abundant source of energy in the environment and solar energy has been used to generate electricity for more than five decades. Attempts have also been made to use solar energy to power CIEDs (Fig. 3d). Therefore, a device containing a 3.24-cm^2^ solar module was used for subcutaneous implantation into a pig to analyze whether transcutaneous solar light could power a pacemaker. The measurements showed an output power of > 3500 µW/cm^2^ at an implantation depth of 2.8–3.84 mm. The output power strongly depended on the implantation depth but successful battery-free VVI pacing could be performed [14]. A 4.6-cm^2^ solar module with an energy buffer implanted subcutaneously in a pig was tested under real-life low-light conditions and was capable of overcoming prolonged periods of darkness for several weeks. The pacemaker continuously paced at a rate of 125 beats per minute for 1.5 months in darkness. The good skin penetrance of infrared light enables sufficient energy harvesting in the subcutaneous solar module to empower the pacemaker even indoors [15]. Another group developed a pacemaker with a subdermal implantable flexible photovoltatic device that generates ~ 647 µW under the skin of mice [32]. However, only long-term studies could demonstrate implications of the body reaction (scaring, encapsulation, blood collection) on the output power. In addition, the effects of parameters such as human clothes, geographic location, daytime or season on energy collection have not yet been determined [15].

## Conclusion

Replacing the batteries of CIEDs with energy harvesters not only extends their lifetime but also allows the devices to be downsized. Deterre et al. [6] developed a micro-cylinder capturing energy from blood pressure variations that is intended to empower leadless pacemakers. Dong et al. [7] successfully tested a minimized lead motion energy harvester that fits into the lead space between wire and silicone coating.

Another option is the biological pacemaker, which is designed to improve the automaticity of the heart by transmitting surrogates for the sinoatrial node, but clinical trials are still pending [3].

Alternative energy harvesting devices still require some improvements in terms of power delivery, long-term stability and energy storage [44].

CIEDs need to fulfil more requirements for diagnostic and telemetric functions, leading to higher energy requirements. Ongoing miniaturization and improved sensor technologies will help to in the development of new devices.

## Declarations

For this article no studies with human participants or animals were performed by any of the authors. All studies mentioned were in accordance with the ethical standards indicated in each case.
